# eHealth tools use and mental health: a cross-sectional network analysis in a representative sample

**DOI:** 10.1038/s41598-024-55910-z

**Published:** 2024-03-02

**Authors:** Dominika Ochnik, Marta Cholewa-Wiktor, Monika Jakubiak, Magdalena Pataj

**Affiliations:** 1 Faculty of Medicine, Department of Social Sciences, Academy of Silesia, 40-555 Katowice, Poland; 2 Faculty of Management, Department of Marketing, Lublin University of Technology, 20-618 Lublin, Poland; 3 Faculty of Economics, Institute of Management and Quality Sciences, Maria Curie-Sklodowska University in Lublin, 20-031 Lublin, Poland; 4 Faculty of Political Science and Journalism, Institute of Social Communication and Media, Maria Curie-Skłodowska University, 20-612 Lublin, Poland

**Keywords:** eHealth management, eHealth tool use, Mental health, UTUAT2, Network analysis, Health services, Health policy

## Abstract

eHealth tools usage is vital for health care systems and increased significantly after the COVID-19 pandemic, which aggravated mental health issues. This cross-sectional study explored whether sociodemographic characteristics and mental health indices (stress and symptoms of anxiety and depression) were linked to the behavioral intention to use eHealth tools and eHealth tools usage in a representative sample from Poland using a network approach. Measurements were conducted in March 2023 among 1000 participants with a mean age of 42.98 (18–87) years, with 51.50% women. The measures included the behavioral intention to use eHealth tools (BI) based on the UTUAT2; eHealth tool use frequency (use behavior) including ePrescription, eSick leave, eReferral, electronic medical documentation (EMD), Internet Patient Account (IKP), telephone consultation, video consultation, mobile health applications, and private and public health care use; and the PSS-4, GAD-2, and PHQ-2. Furthermore, sociodemographic factors (sex, age, children, relationship status, education, and employment) were included in the research model. Network analysis revealed that mental health indices were weakly related to eHealth tools use. Higher stress was positively linked with mobile health application use but negatively linked to video consultation use. Use of various eHealth tools was intercorrelated. Sociodemographic factors were differentially related to the use of the eight specific eHealth tools. Although mental health indices did not have strong associations in the eHealth tools use network, attention should be given to anxiety levels as the factor with the high expected influence.

## Introduction

The World Health Organization (WHO)^1^ defines eHealth as the use of information and communication technologies for health and lists eHealth tools, such as electronic health records, patient information systems, and telehealth (telephone and video consultations). According to the European Commission on Digital Health and Care, eHealth tools are critical due to population aging, the unequal quality of and access to health care systems, and shortage of health care professionals^2^. However, definitions of eHealth may vary depending on specific contexts or elements, most frequently focusing on communicative aspects. Nevertheless, there are at least 36 definitions of eHealth^3^. eHealth is called “ubiquitous health” and is a dynamic network of interconnected systems^4^. During the coronavirus disease 2019 (COVID-19) pandemic, eHealth tools became crucial for health care systems, and their use significantly increased^5^. Therefore, it is vital to explore eHealth tools use, the intercorrelations of eHealth tools use, and the current sociodemographic and mental health characteristics of eHealth users. This will allow us to evaluate possible barriers to and facilitators of eHealth tool use in the current postpandemic context.

A review of sociodemographic factors related to eHealth tools usage showed high complexity and diversity. Overall, younger age, higher education level, and living with others were related to more frequent eHealth tool use, regardless of employment status. Furthermore, the results regarding gender and place of residence were inconsistent^6^. On the one hand, research has shown that men are less likely to engage in eHealth tool use than women^7–11^. In contrast, other studies have shown no significant relationship between gender and eHealth tool use^12–14^. People living in rural areas have less access to eHealth tools and worse economic situations but are simultaneously in urgent need of eHealth tools due to restricted availability of in-person medical services^6^. However, previous studies have shown that living in rural versus urban areas does not lead to differences in telemedicine use^15^. Age is negatively linked to eHealth tool use and searches for health-related information^7,12,16–18^. During the COVID-19 pandemic, older people used video consultations significantly less frequently than younger people^11,19^. However, some research has shown that there is no significant relation between age and eHealth tool use^20^.

The literature emphasizes the positive association between employment status and eHealth tool use; however, retired participants used eHealth tools in a similar manner to employed participants^21^. Little is known about how marital or relationship status are associated with eHealth tool usage, except for living arrangements, with a study showing that patients with chronic diseases who live with others used eHealth tools more frequently due to social support than patients living alone^6^. Marital or relationship status should be included in eHealth tool use models, as it is crucial for health outcomes^22^.

Access to eHealth tools varies by tool type and the sociodemographic characteristics of potential users. Therefore, disparities in telemedicine use (e.g., telephone and video consultation) may not be transferable to patient portal use^23^.

E-mental health research has expanded and has often focused on remote treatment or smartphone-based mental health interventions for depression and anxiety disorders^24–26^. However, research on mental health indices as characteristics of eHealth users is scarce.

## The present study

This study aimed to identify the network of eHealth tools use frequency in the postpandemic period. We aimed to explore the relations between a variety of eHealth tools use (use behavior), behavioral intention for eHealth tools use, type of health care use (private, public), sociodemographic characteristics, and mental health indices (stress and symptoms of anxiety and depression) of users, using the network analysis approach.

The present research was conducted in the Polish context. The COVID-19 epidemic was declared over in July 2023^27^, and during the data collection period (March 2023), most of the pandemic restrictions had been waved except for the requirement of wearing masks when visiting medical services. It is also vital to note that the majority (88.40%) of Polish citizens have access to the internet, and the number of mobile phones exceeds the population size (127.70% vs. population)^28^. Therefore, access to eHealth is available in Poland.

During the period of improvements in pandemic severity (May/June 2021), the prevalence rates of perceived stress, anxiety, and depression were still high in Poland, at 86.55%, 38.12%, and 42.15%, respectively^29^. Although perceived stress significantly dropped from a starting point of 90.36%^30^, mental health issues were still ubiquitous. Therefore, it is crucial to explore the relationships among mental health indices, behavioral intention to use eHealth tools and use behavior. However, little is known in this area.

The present study was partially based on the unified theory of acceptance and use of technology (UTUAT)^31^, originally developed regarding employee acceptance of technology in organizations. This theory was later extended (UTUAT2)^32^ to use in other contexts. It has already been used to develop a generalized adoption model for mobile health^33,34^. We examined the relationship between behavioral intention to use eHealth tools and use behavior, measured by the frequency of use of eight eHealth tools (ePrescription, eSick leave, eReferral, electronic medical records [EMR], the Internet Patient Account [IKP], phone and video consultations, and mobile health applications). The choice of specific eHealth tools was based on the latest review of the Polish health care system^35^. Other than sociodemographic factors established to moderate behavioral intentions, such as age and gender^32^, a literature review suggested that place of residence (density), education level, relationship status, living arrangements, presence of children, and employment status should also be added to the model. We expected to find direct relations of sociodemographic factors with behavioral intentions and use behavior. In previous research, an additional psychological factor, quality of life, was included in the model; quality of life was related to use behavior but not to behavior intention^34^. Anxiety and depression were important in the model but for acceptance of e-mental health interventions rather than for factors directly related to use behavior^36^. Furthermore, in technology acceptance model research, anxiety often refers to personal safety and privacy related to new technology use^37^, such as mobile anxiety, technology anxiety, or computer anxiety^38^.

Moreover, we included the frequency of use of private and public health care. Current individual spending on private health care is constantly increasing. Only one in four Poles has a good opinion of the quality of public health care. Poles are much more convinced by the private medical sector^39^. Some eHealth tools are inseparable from the public health sector (e.g., IKP); however, some can relate to the private sector (e.g., phone and video consultations or mobile health applications). Therefore, we aimed to explore relations between the type of healthcare sector (public and private) and the use of eHealth tools, as this context has not been investigated yet to the authors’ knowledge.

**Figure 1:**
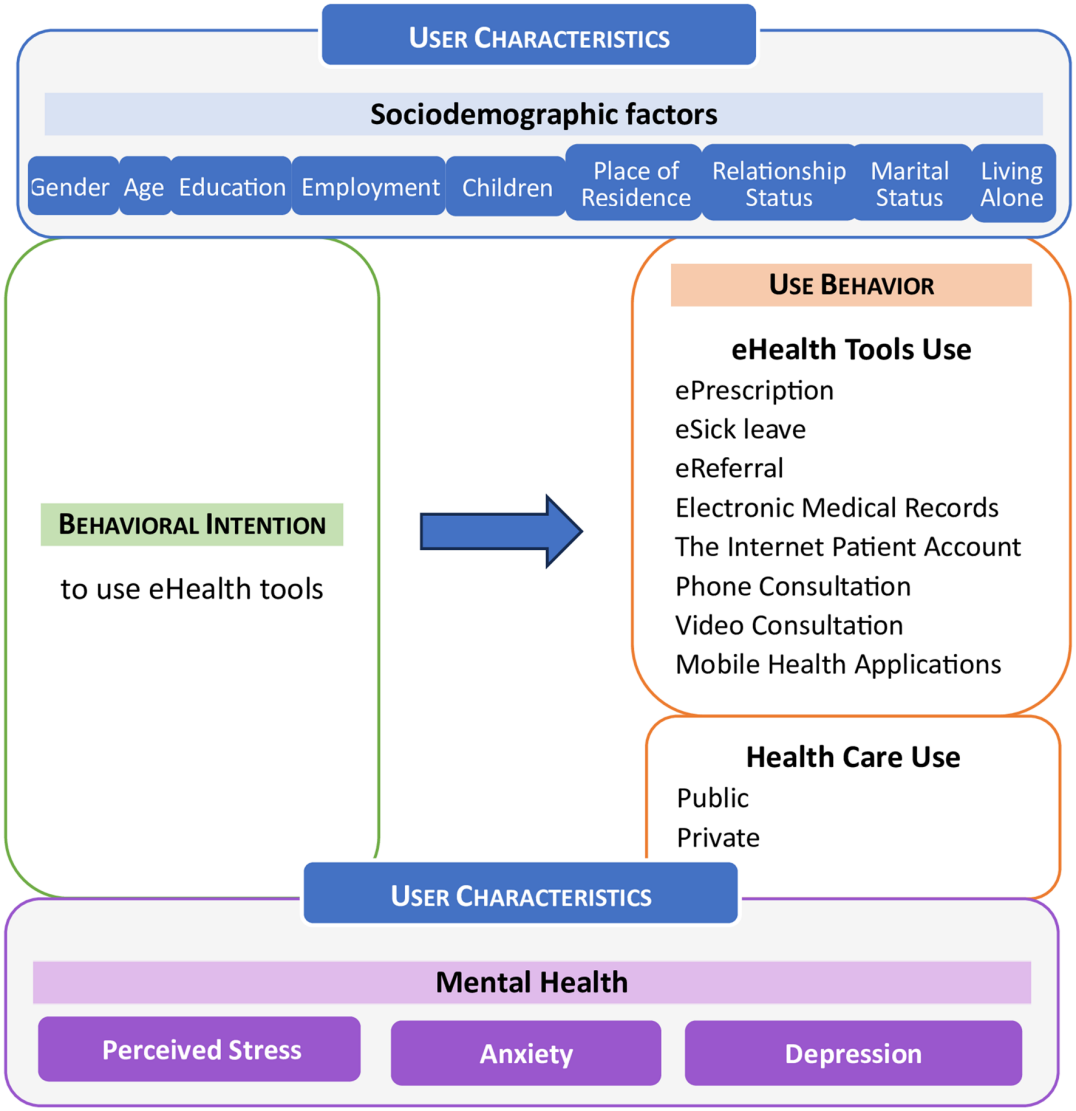
Theoretical model for the eHealth tools use in the general population. Own preparation, partially based on the UTUAT2 model^32^, adapted to eHealth tools use and with extended user characteristics to mental health.

Therefore, this is the first study to explore the relations between detailed use behavior (measured by the use frequency of eight eHealth tools), behavioral intention to use eHealth tools, mental health indices (perceived stress, anxiety, and depression), and a variety of sociodemographic factors (age, gender, place of residence, education level, relationship status, living arrangements, presence of children, and employment status) using a network approach among a representative Polish sample of 1000 adult participants (over 18 years of age). The assumptions for the theoretical model for the eHealth tools used in the general population are presented in Fig. 1.

The use of the network analysis was due to the explorative character of the research. A network approach can be used to explore the structure of relationships among variables in the absence of a robust theory regarding these relationships^40^. Network models are also increasingly applied in the field of health psychology^41^. Furthermore, a network theory of mental health disorders has been proposed by Borsboom^42^, highlighting the role of direct interactions between specific symptoms. However, scale-level mental health indices are also used to explore risk and protective factors in the network approach^43^. This approach was utilized in our study. A detailed description of the network approach is presented in the Methods section.

## Results

### Participants

The sample size was 1000 participants, and the sample was representative of the Polish population. All participants signed consent forms and were eligible to participate in the study (over 18 years of age). No answers were omitted. The age ranged from 18 to 87 years, and the median age was 41 years. All participants declared themselves to be women or men. There were more women than men (52%), although this difference was not significant. Most participants were 35–44 years old (25%) and lived in urban areas (over 20,000 residents) (68%). Educational level was equally distributed. Most of the participants were married (53%), in a relationship (78%), living with others (89%), and employed (74%) and had children (70%). Since the proportions of married versus unmarried participants greatly differed, this variable was excluded from further statistical analysis, and only relationship status was included. Descriptive statistics and Pearson’s χ^*2*^ test results are presented in Table 1.

**Table 1:** Sociodemographic characteristics of the sample.

Variable	Total (*N* = 1000)				
	*N*	%	*M*	*SD*	χ^*2*^
Gender					1.16
Man	483	48.30			
Woman	517	51.70			
Age, years (18–87)			42.98	14.54	107.95***
18–24	110	11.00			
25–34	212	21.20			
35–44	254	25.40			
45–54	185	18.50			
55–64	132	13.20			
Over 65	107	10.70			
Place of residence				68.49***	
Village	216	21.60			
Small town	99	9.90			
Town	260	26.00			
City	253	25.30			
Big city	172	17.20			
Education level					9.22**
Secondary education or lower	548	54.80			
Tertiary education and higher(BA, MA, PhD)	452	45.20			
Education level					845.20***
Married	534	53.40			
Divorced	51	5.10			
Cohabitating	243	24.30			
Widowed	25	2.50			
Never married	147	14.70			
Relationship status				306.92***	
Single	223	22.30			
In a relationship	777	77.70			
Living arrangements					
Living alone	107	10.70			617.80***
Living with others	893	89.30			
Have children					161.60***
Yes	701	70.10			
No	299	29.90			
					238.14***
Employed	744	74.40			
Unemployed	256	25.60			

*M* = mean; *SD* = standard deviation.

**p < 0.01, and ***p < 0.001.

**Figure 2:**
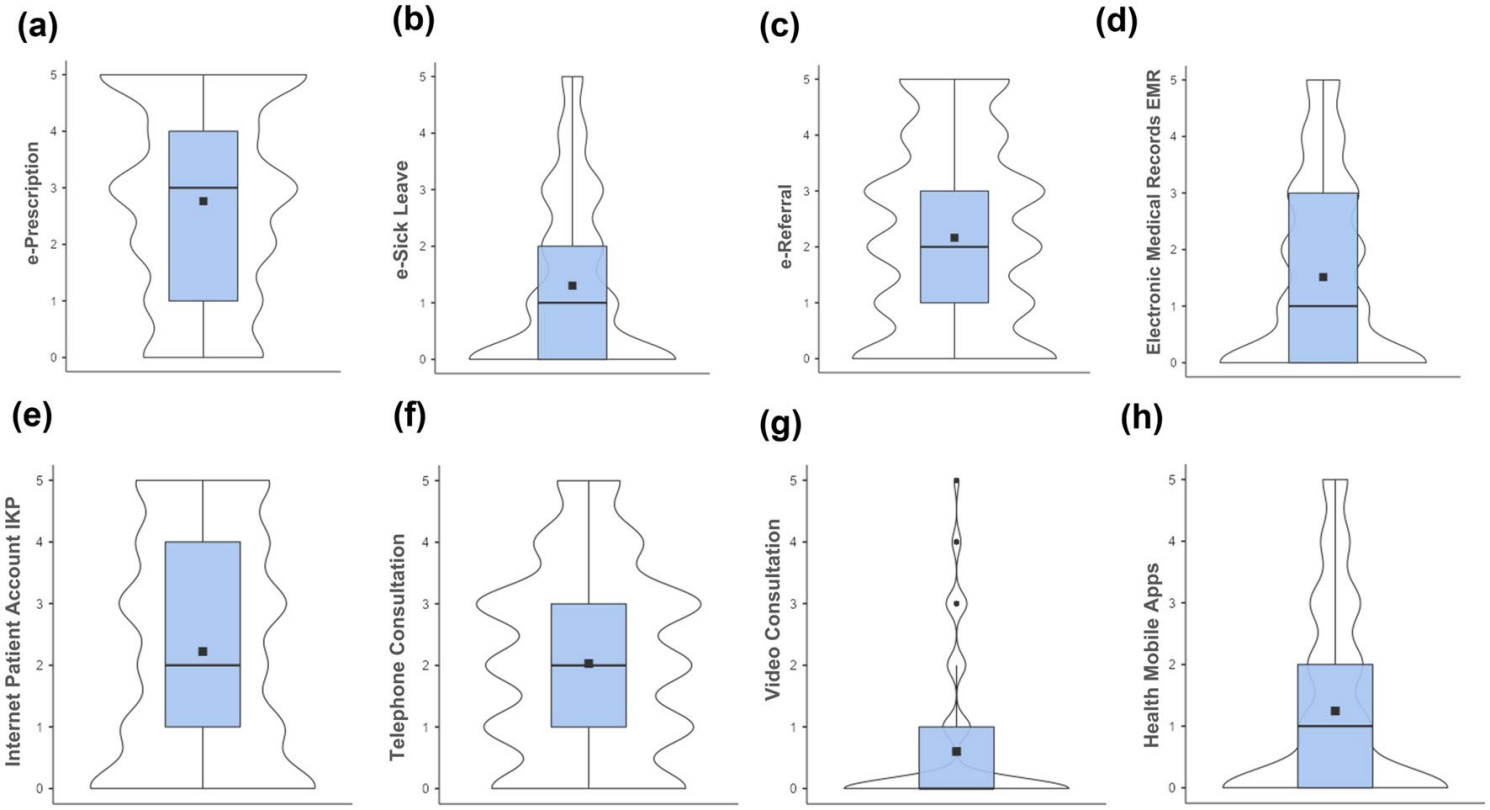
Distribution and mean scores of eHealth tool use frequency: (**a**) ePrescription, (**b**) eSick leave, (**c**) eReferral, (**d**) electronic medical records (EMRs), (**e**) the Internet Patient Account (IKP), (**f**) telephone consultation, (**g**) video consultation and (**h**) mobile health applications among a representative population in Poland (*N* = 1000), displayed with violin plots and box plots. Mean scores are represented by squares and outliers by dots. The frequency scale was from 0 = never to 5 = very often.

Regarding eHealth tool use frequency, video consultation was hardly ever used (*M* = 0.6, *SD* = 1.18), while ePrescription (*M* = 2.76, *SD* = 1.72) was the most frequently used tool in the representative sample. Details on the distributions, mean scores, and outliers are shown in Fig. 2.

**Figure 3:**
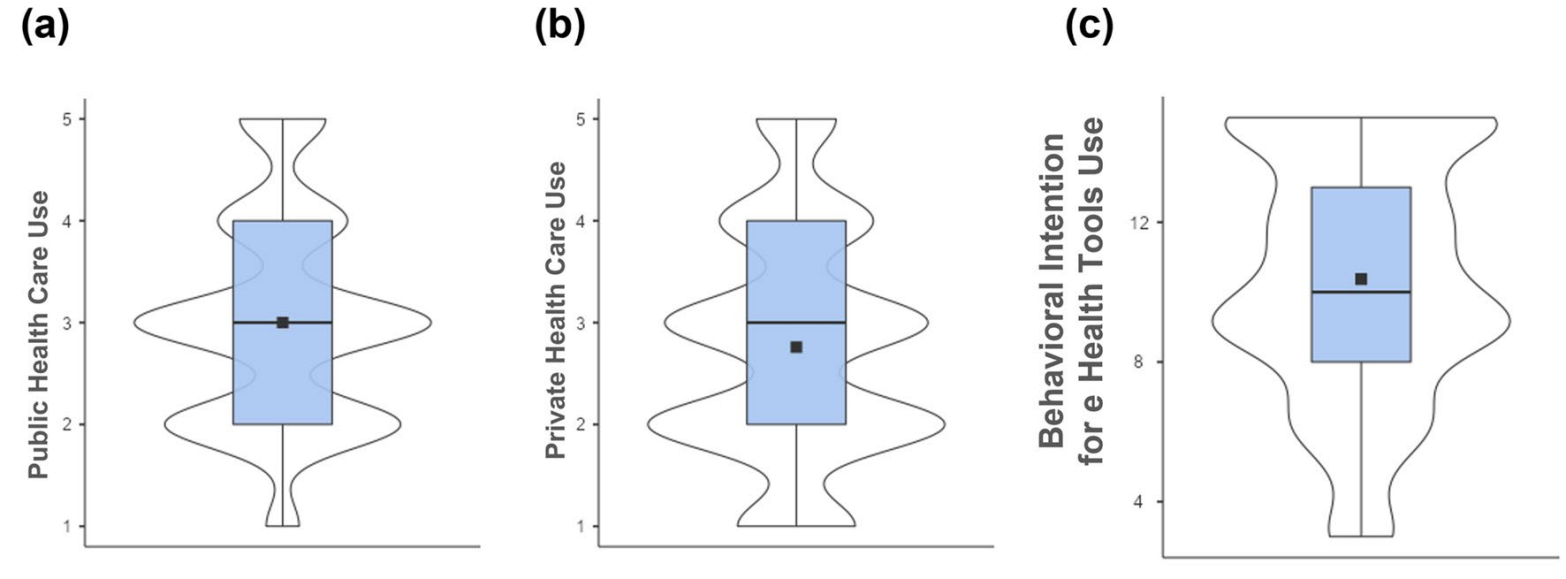
Distribution and mean scores of (**a**) public health care use, (b) private health care use frequency, and (**c**) behavioral intention to use eHealth tools among a representative population in Poland (*N* = 1000), as shown in violin plots and box plots. Mean scores are represented by squares. The frequency scale was from 1 = never to 5 = very often.

Descriptive statistics regarding public and private health care use frequency are presented in Fig. 3.

**Figure 4:**
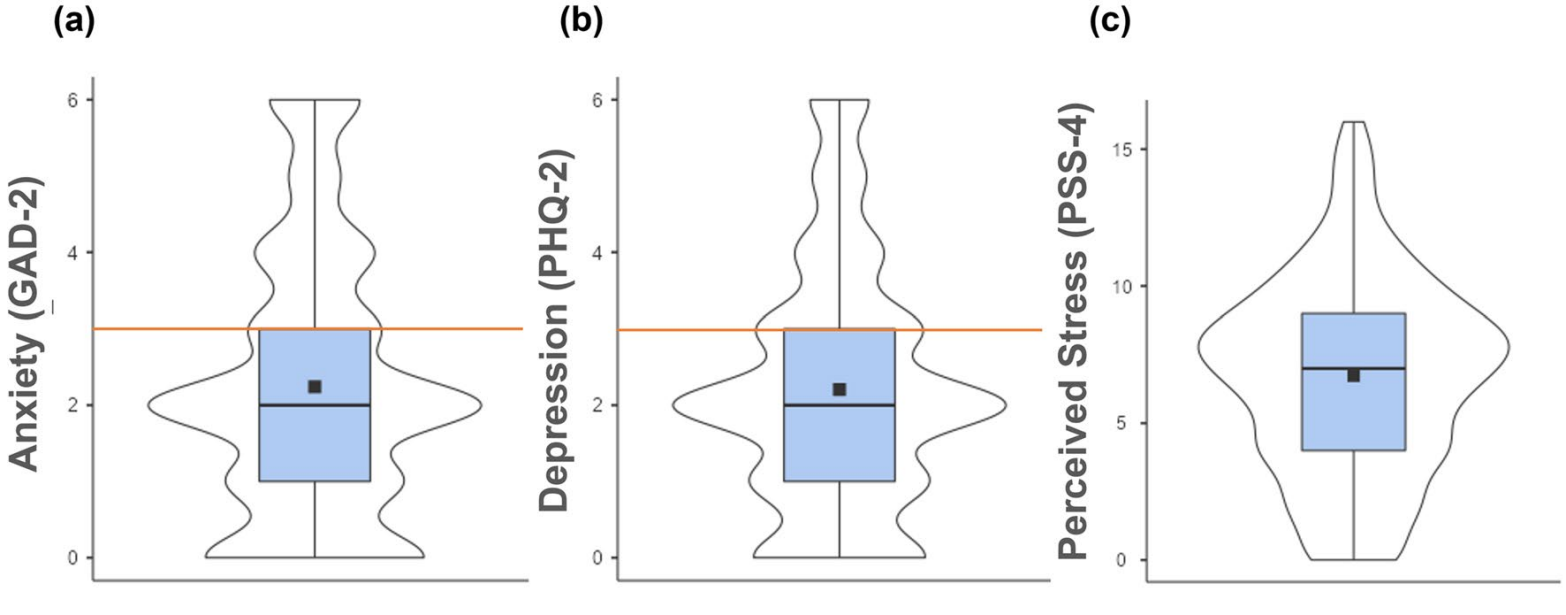
Distribution and mean scores of (**a**) anxiety (GAD-2); (**b**) depression (PHQ-2); and (**c**) perceived stress (PSS-4) among a representative population in Poland (*N* = 1000), as shown with violin plots and box plots. Mean scores are represented by squares and outliers by dots. The orange line represents the cutoff score.

Descriptive statistics revealed that the majority of participants scored below the cutoff score for anxiety and depression symptoms. However, most of the research sample had high scores (over 6 points) on the Perceived Stress Scale (*M* = 6.73, *SD* = 3.58). Details are presented in Fig. 4.

### Proportions of eHealth tools use, behavioral intention, and mental health conditions prevalence

Regarding eHealth tools usage, the highest proportion of respondents had used at least once ePrescription (86.30%), telephone consultation (80.60%), or eReferral (78.00%), although most had never used a video consultation (73.90%). More than half of the participants had used eSick leave (55.60%) and electronic medical records (60.10) at least once. Mobile health application use was equally distributed, with 50.10% of participants using this tool.

Almost all participants used public health care (95.80%), while a slightly smaller proportion used private health care (87.10%).

The behavioral intention to use eHealth tools was evenly distributed, and 47% of participants reported a high intention to use these tools.

The prevalence rates of anxiety, depression, and perceived stress were 34.90%, 34.80%, and 64.60%, respectively. Comorbidities occurred in 24.50% of the study sample. However, the majority of participants had no anxiety or depression symptoms (54.80%). All details are presented in Table 2.

**Table 2:** Prevalence rates of e-Health tools use, health care use, behavioral intentions to use eHealth tools, and mental disorders risk according to the χ^2^ test.

Variable	All participants *N* = 1000		
	*n*	%	χ^2^
ePrescription			527.08***
Never used (0)	137	13.70	
Used (1)	863	86.30	
eSick leave			12.54***
Never used (0)	444	44.40	
Used (1)	556	55.60	
eReferral			313.60***
Never used (0)	220	22.00	
Used (1)	780	78.00	
Electronic medical records (EMRs)			40.80***
Never used (0)	399	39.90	
Used (1)	601	60.10	
Internet Patient Account (IKP)			262.14***
Never used (0)	244	24.40	
Used (1)	756	75.60	
Telephone consultation			374.54***
Never used (0)	194	19.40	
Used (1)	806	80.60	
Video consultation			228.48***
Never used (0)	739	73.90	
Used (1)	261	26.10	
Mobile health applications			0.004
Never used (0)	499	49.90	
Used (1)	501	50.10	
Public health care use			839.06***
Never used (0)	42	4.20	
Used (1)	958	95.80	
Private health care use			550.56***
Never used (0)	129	12.90	
Used (1)	871	87.10	
Behavioral intention to use eHealth tools (BI score ≥ 11)			3.36
Low (0)	529	52.90	
High (1)	471	47.10	
Anxiety symptoms (GAD-2 score ≥ 3)			91.20***
No (0)	651	65.10	
Yes (1)	349	34.90	
Depression symptoms (PHQ-2 score ≥ 3)			92.42***
No (0)	652	65.20	
Yes (1)	348	34.80	
Anxiety × depression symptoms			209.53***
No Risk (0)	548	54.80	
One Disorder Risk (1)	207	20.70	
Comorbidity Risk (2)	245	24.50	
Perceived stress (PSS-4 score ≥ 6)			85.26***
Low (0)	354	35.40	
High (1)	646	64.60	

### Correlations

**Figure 5:**
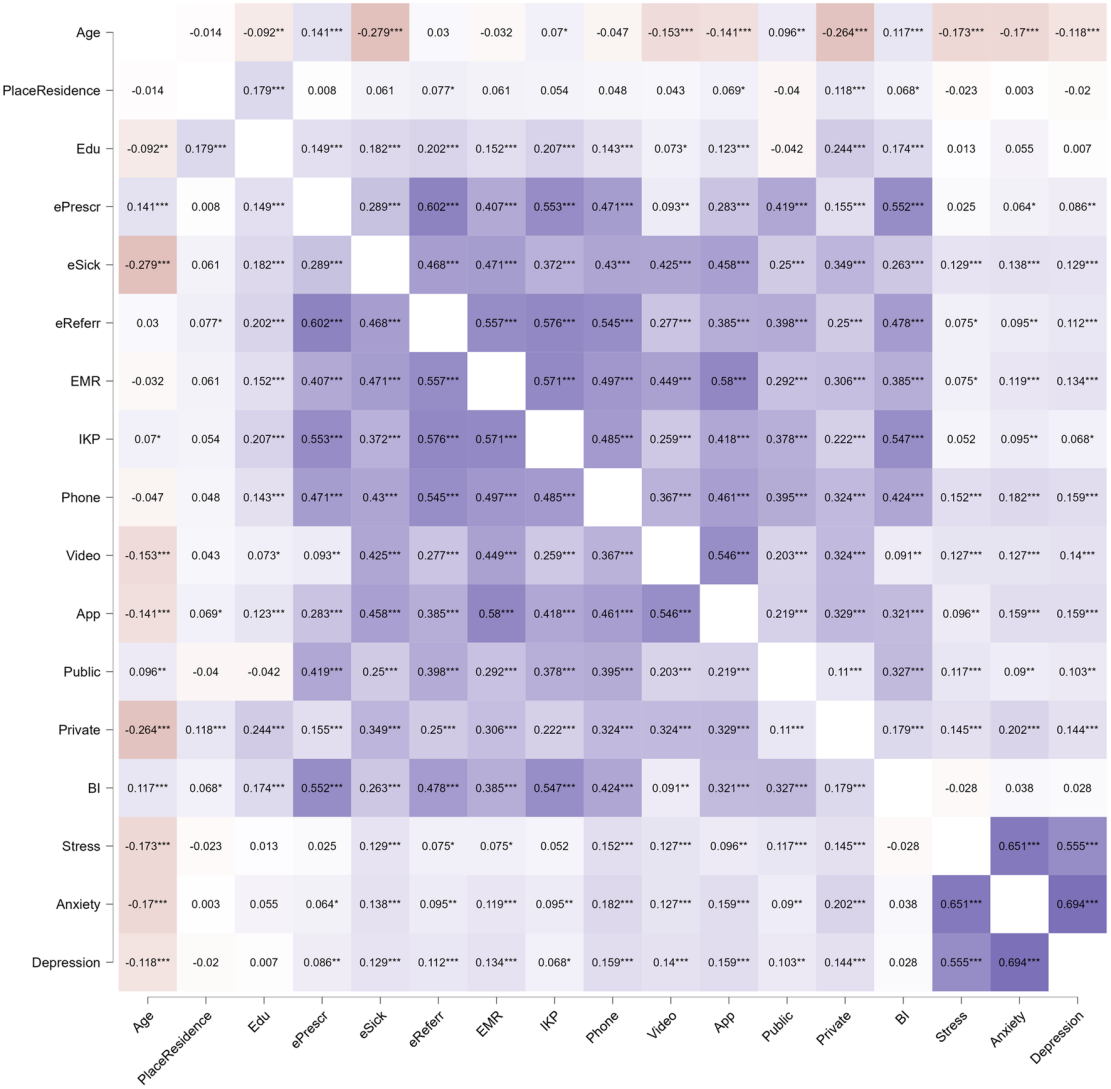
Spearman’s *rho* heatmap. *N* = 1000; ePresr, ePrescription; eSick, eSick leave; eReferr, eReferral; EMR, electronic medical records; IKP, Internet Patient Account; Phone, telephone consultation; Video, video consultation; App, mobile health applications; Public, public health care use; Private, private health care use; BI, behavioral intention to use e-Health tools. Purple represents positive correlations, while orange represents negative correlations. The most intense shades represent a large effect size, while the lightest shades represent a small effect size. *p < 0.05, **p < 0.01, ***p < 0.001.

In the first step, Spearman correlation analysis was performed to examine relationships among sociodemographic characteristics, eHealth tools use, health care use, behavioral intention to use eHealth tools, and mental health indices. All details are presented in the heatmap in Fig. 5. Description of the correlations is presented in the Supplementary Materials.

### Differences according to sociodemographic characteristics

A Mann‒Whitney U test was performed to evaluate whether eHealth tool and health care use differed according to gender, relationship status, living arrangements, presence of children, and employment status.

Women used telephone consultations more frequently and less frequently video consultations compared to men. Women also used private health care more often than men. Participants in a romantic relationship more frequently used most eHealth tools and public and private health care and had a higher BI than single participants. Participants living with others used eSick leave more frequently than those living alone. Since living arrangements had a significant but very small difference in only one eHealth tool, this variable was excluded from further statistical analysis. Participants with children used eHealth tools such as eSick leave, eReferral, EMR, and the IKP significantly more often than childless participants. Employment status significantly differentiated eHealth tools use, health care use, and BI. Employed participants had higher use frequency compared to unemployed participants.

A detailed description of the differences is presented in the Supplementary Materials.

### Network analysis

**Figure 6:**
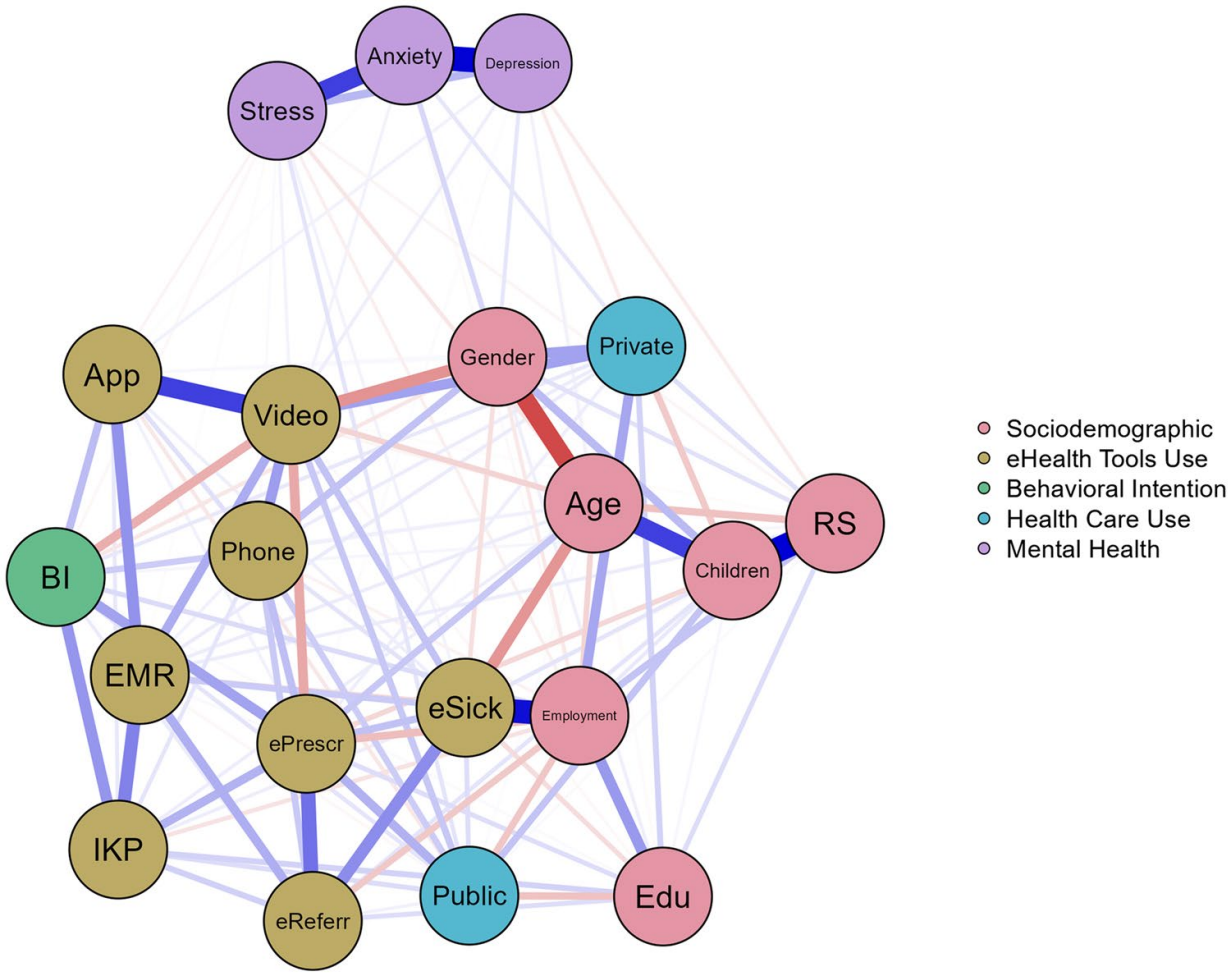
Network of eHealth tool use frequency including health care use, behavioral intentions, and sociodemographic characteristics (*N* = 1000). Nodes represent the following variables: ePresr, ePrescription; eSick, eSick leave; eReferr, eReferral; EMR, electronic medical records; IKP, Internet Patient Account; Phone, telephone consultation; Video, video consultation; App, mobile health application. Health care use: Public, public health care use; Private, private health care use frequency; BI, behavioral intention to use eHealth tools. The dichotomized sociodemographic nodes are as follows: Gender (women = 1), Children (presence of children, yes = 1), RS = relationship status (in a relationship = 1), Education (tertiary education and higher = 1), Employment (employed = 1). Blue edges represent positive relations; red edges represent negative relations. The thickness of the edges represents the strength of the relations between the nodes.

Based on correlation analysis and group comparisons, variables significantly associated with eHealth tool use frequency were included in the network model. The included variables in the eHealth tools use network model were gender, age, educational level, private and public health care use, and mental health indices. Place of residence and living arrangements were excluded from the model. There were 20 nodes, the number of nonzero edges was 138/190, and the sparsity was 0.27. The estimated network was interconnected. After controlling for all variables in the network, a visualization of relationships (edges) among nodes was generated. The eHealth tools use model constructed using the network approach is presented in Fig. 6.

The network analysis showed positive relationships between nodes denoting eHealth tools use. However, video consultation use was inversely related to ePrescription use. The most robust edges of eHealth tool use were between video consultation and mobile health application use, between EMR and IKP use, between ePrescription and eReferral use, and between eReferral and eSick leave use.

Among sociodemographic characteristics and frequency of using eHealth tools, the strongest edge was between employment status and eSick leave use. Age was positively related to ePrescription use but negatively related to eSick leave use. Lower education level, presence of children, and unemployment were related to public health care use, while being childless and employed were related to private health care use. Edges associated with BI were weak. Male sex, older age, higher education level, and employment were linked to BI. BI was positively related to ePrescription, IKP, phone consultation, and mobile health app use but negatively related to video consultation use.

Edges between eHealth tools use and mental health indices were weak. Higher stress was linked to more frequent eSick leave, phone and video consultation use and less frequent use of ePrescription and mobile health apps. Anxiety symptoms were positively linked to phone consultation use, while higher depression symptoms were associated with more frequent video consultation and mobile health app use. Edges between mental health and health care use were also weak. Higher stress was linked to public health care use, while higher anxiety was linked to private health care use frequency. The exact weight matrix is presented in Supplementary Table S1.

### Network centrality nodes

The centrality of nodes indicates their relative importance in the context of the other nodes in the network^44^. Node strength indicates direct connections to other nodes (based on the weighted number and strength of all edges of the node linked to other nodes). The highest node strength in the estimated network was observed for employment status, video consultation use, eSick leave use, ePrescription use, and age.

**Figure 7:**
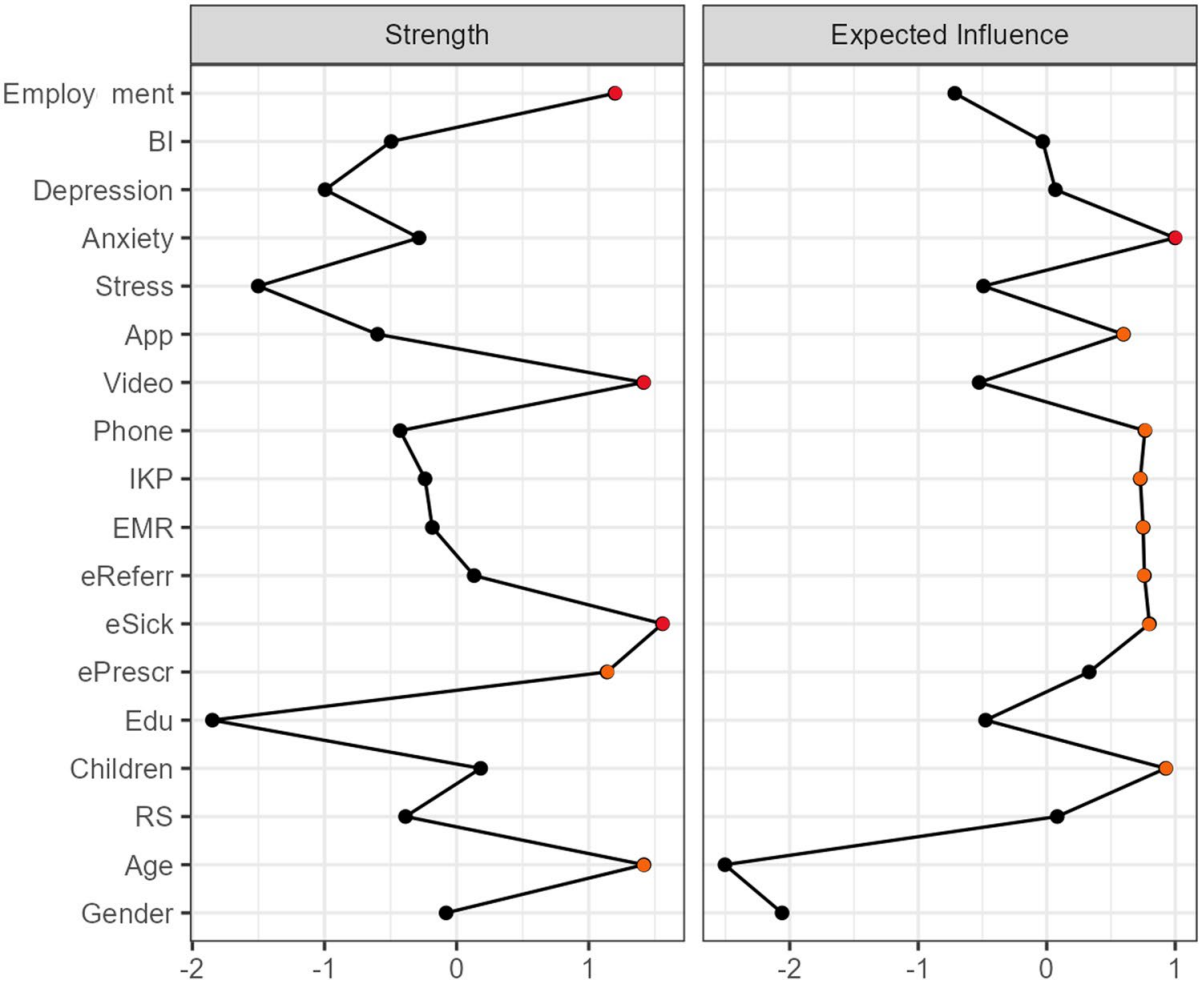
Standardized centrality and expected influence values nodes in the estimated network. Red dots denote estimations over 1, and orange dots denote estimations between 0.5 and 1. ePresr, ePrescription; eSick, eSick leave; eReferr, eReferral; EMR, electronic medical records; IKP, Internet Patient Account; Phone, telephone consultation; Video, video consultation; App, mobile health applications; Public, public health care use; Private, private health care use; BI, Behavioral intention to use e-Health tools; Edu, Education; RS, Relationship Status.

Expected influence (EI) is a centrality measure that reflects a node’s importance in terms of activating or deactivating other nodes in a network that has negative edges^45^. Anxiety symptoms had the greatest influence, and further influential nodes were eHealth tools (mobile health app, phone consultation, IKP, EMR, eReferral, and eSick leave use) and the presence of children. Therefore, nodes high in centrality strength differed from nodes high in expected influence. Details regarding node centrality are presented in Fig. 7. Additional centrality measures (betweenness and closeness) are presented in Supplementary Table S2. Clustering measures per variable are presented in Supplementary Table S3.

### Network accuracy

**Figure 8:**
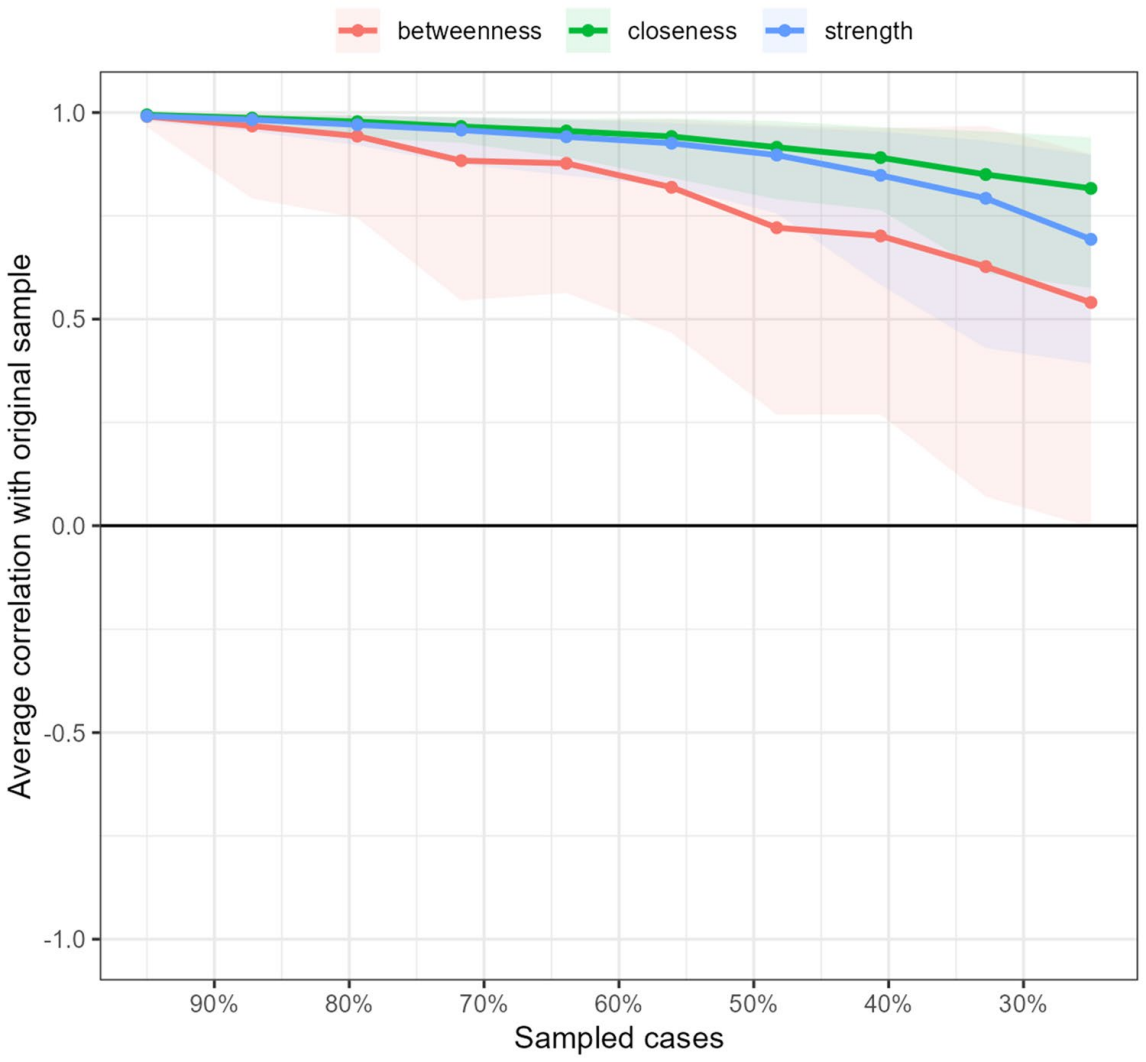
Stability of the centrality indices (betweenness, closeness, and strength) in the estimated network. The shaded area indicates 95% confidence intervals of correlation estimates resulting from 1000 bootstraps.

The accuracy of the centrality indices, determined using a subset bootstrap of 1000, was very good. Better stability is indicated by higher values of centrality estimates. The correlation stability (CS) coefficient was over 0.5. Details regarding the case-dropping bootstrap analysis, which estimated average correlations between centrality indices in the total sample and centrality indices in a random subsample, retaining only a certain portion of cases (90%-100%), are presented in Fig. 8.

**Figure 9:**
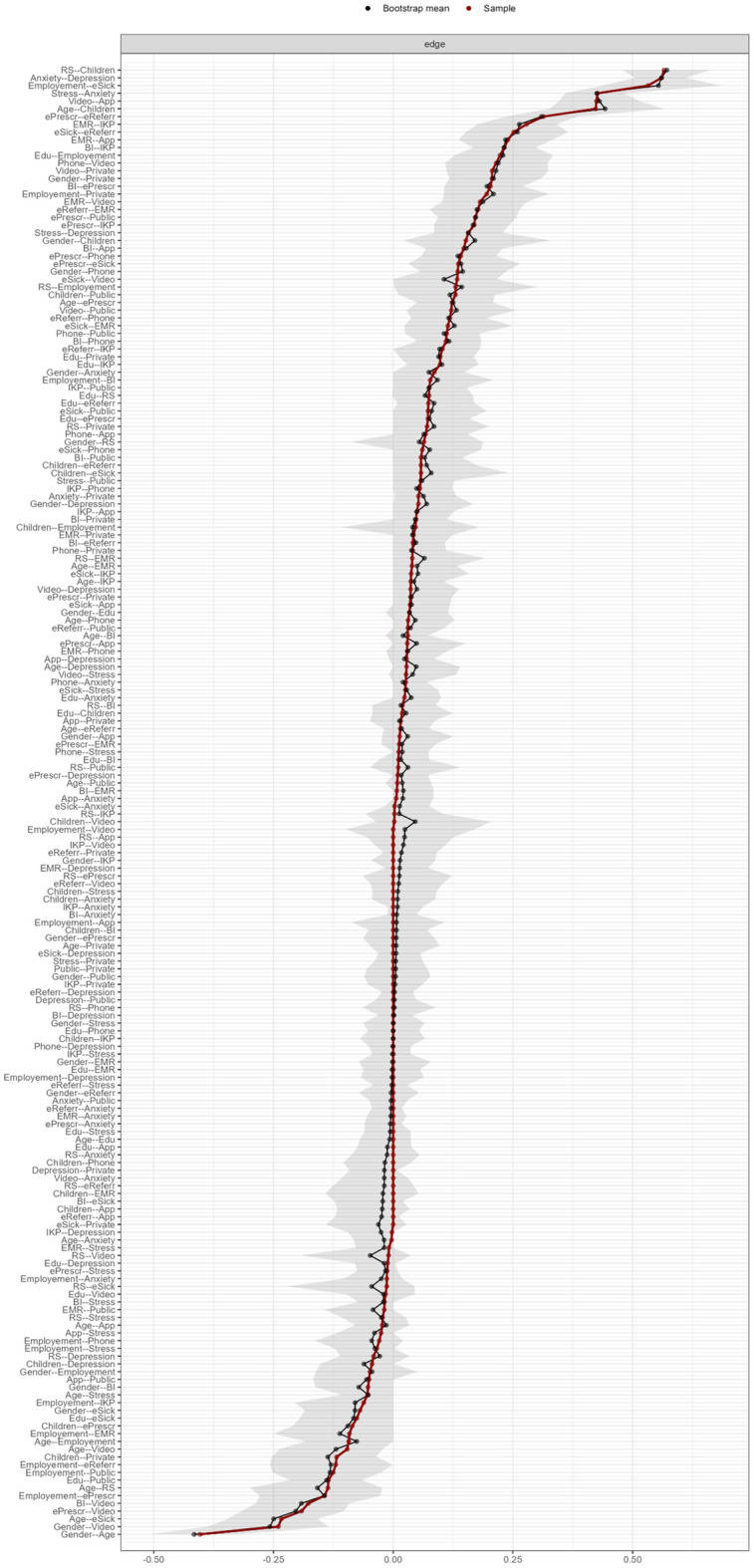
Representation of robustness and stability of the edge weight estimates. The red line represents sample values, the black line represents bootstrapped means, and the shaded area represents 95% confidence intervals resulting from 1000 bootstraps.

Edge stability was calculated with 1000 nonparametric bootstrap samples for edge-weight estimation. The accuracy of edge weights estimated by 95% confidence intervals (CI) for these estimates showed good stability. The widest range of 95% CIs was for edge weights on the edges between sociodemographic nodes. Edge stability data are presented in Fig. 9.

## Discussion

This was a unique study of eHealth tool use according to sociodemographic characteristics, mental health indices, type of health care, and BI, in a nationally representative sample of 1000 Polish citizens.

We showed the prevelance of eHealth tools use and confirmed the theoretical model for the eHealth tools use in the general population using network analysis.

### Prevalence of eHealth tools use and type of health care use

The most commonly used eHealth tools were ePrescription (86.30%), telephone consultation (80.60%), eReferral (78.00%), and the Internet Patient Account (IKP) (75.60%). Approximately half of the participants used eSick leave (55.60%), EMR (60.10%), and mobile health applications (50.10%) at least once. The least frequently used eHealth tool was video consultations (26.10%). Such a high proportion of eHealth tool use (e.g., of ePrescription) may be due to the general increase in eHealth tool availability during the COVID-19 pandemic^5^.

Nearly half of the participants (47.10%) reported that they had high behavioral intention to use eHealth tools. Most of the participants used public (95.80%) and private health care (87.10%), indicating that a public health care system is insufficient.

Public health care (95.80%) and private health care (87.10%) were widely used. This means that public health care is insufficient, and patients often used paid services. It can also result from access to paid medical services through companies as part of the employee benefits package.

The prevalence rates of high perceived stress, anxiety symptoms, depression symptoms, and depression comorbidity symptoms were 64.60%, 34.90%, 34.80%, and 24.50%, respectively. High perceived stress and depression symptoms were significantly less prevalent than during the COVID-19 pandemic period in February 2021, when high perceived stress, anxiety symptoms, and depression symptoms had prevalence rates of 90.36%, 37.44% and 42.83%, respectively^29^. In the period of reduced pandemic severity in May/June 2023, the prevalence rates of high perceived stress (86.55%) and depression symptoms (42.15%) were also higher, while that of anxiety was similar (38.12%)^29^ to the present study.

The significance of differences in sociodemographic factors showed that women used telephone and video consultations and private health care more often than men. Moreover, being in a romantic relationship, having children, and being employed increased the use of eHealth tools.

### Network analysis of eHealth tools use

The exploration of the research model revealed that sociodemographic factors were differentially related to the use of the eight specific eHealth tools. Mental health was weakly related to eHealth tool use and BI. Only perceived stress was linked to both BI and eHealth tools use. Higher stress was associated with lower BI and less frequent mobile health application use but more frequent video consultation use. Conversely, a higher BI was related to more frequent mobile health application use and less frequent video consultation use. The most influential node in the estimated network was anxiety symptoms. The strongest nodes were age and employment status, along with video consultation, eSick leave, and ePrescription use.

Based on the UTUAT model^31,32^, we assumed a positive relationship between behavioral intentions and use behavior. Higher behavioral intentions were strongly linked to more frequent ePrescription and IKP use and to more frequent mobile health application and phone consultation use. The positive relationship with eReferral was weak. No such relationship with BI was found for video consultation or eSick leave use. Communication regarding eSick leave was between the employer and health care system; thus, eSick leave users were less engaged and active in the intentional use of this tool. This might be the reason for the lack of association with BI. However, unexpectedly, there was a negative relationship between BI and video consultation use. A higher BI was linked to lower video consultation use. This might be due to the lower frequency of use of this eHealth tool in the sample.

BI was not associated with anxiety and depression symptoms but was negatively linked to stress. Higher perceived stress was related to lower BI and lower mobile health application use. Therefore, stress can hinder BI and mobile health application use; however, the effect size was small. In contrast, higher stress was linked to more frequent video consultation use, which in turn was linked to lower BI.

The use frequency of eHealth tools was positively correlated in the network. The only negative relation was between the most commonly used tool, ePrescription, and the least commonly used tool, video consultation. Use of ePrescription was linked to more frequent use of eSick leave, the IKP, and phone consultations. The strongest relation was between ePrescription and eReferral use. Participants who used video consultation more often also used eSick leave, EMR, and phone consultations. The strongest relationship between eHealth tools was between video consultation and mobile health application use. We assume that these tools are linked to internet competency/literacy. Mobile health application usage was also linked to more frequent EMR usage. Frequent eSick leave use was linked to eReferral and EMR use. Additionally, eReferral use was linked to more frequent EMR, IKP, and telephone consultation use. The more frequently participants used EMRs, the more often they used the IKP. Within the eHealth tools network, the most interconnected tools (5 edges each) were ePrescription, eReferral, and EMR use. Therefore, these eHealth tools are crucial for the use of other eHealth tools.

The relationship between mental health and eHealth tools use frequency was positive but weak. Higher stress and depression symptoms were linked to more frequent video consultation use. This may be due to the perceived urgency of the need and search for (virtual) in-person contact. Video consultation, even though the least popular eHealth tool, most closely approximates in-person contact, as it contains verbal and nonverbal communication. On the other hand, depression symptoms were related to more frequent mobile health application use. Anxiety symptoms were linked to more frequent telephone consultations and private health care use. Overall, worse mental health was associated with more frequent usage of some medical services and therefore with pro-health behaviors. The small effect sizes might be due to missed behaviors of participants with mental health issues. Nevertheless, these participants more frequently chose eHealth tools relating to human interaction.

The relationships between sociodemographic characteristics with eHealth tools use were diverse. More frequent use of ePrescription was linked to older age and unemployment. This means that ePrescription, as the most widely used eHealth tool (along with telephone consultation), is easily accessible to older people and, therefore, widely used. This contrasts with previous findings^7,12,16–18^. On the other hand, ePrescription use does not require eHealth literacy and, thus, is also more accessible for older users.

eSick leave was commonly used by younger and employed people, as it is a tool related to employment status. eReferral, which involves the need to visit a specialist, was weakly related to sociodemographic factors. Older and more educated participants with children more frequently used eReferral, but those relationships were very weak. However, eReferral was also related to retirement. In this case, it could refer to participants who did not work due to age. The relationships of sociodemographic factors with EMR and IKP use were very weak. Gender was linked to telephone and video consultation use. Women more often used phone consultations but less frequently used video consultations than men. These findings are in line with previous research on technology use. Women tend to choose interpersonal communication^46,47^ but are less eager to show their appearance online^48^.

In a previous analysis, living alone was associated with a lower probability of eHealth tool use among patients with chronic disease^6^. Our research showed only one very small difference in eSick leave use, with people living alone using this eHealth tool less frequently than those living with others. This lack of difference might be due to the representative sample. Relationship status was hardly related to eHealth tool use, with participants in a relationship using only one tool (EMR) more often than single participants. There were significant differences in eHealth tools use due to relationship status; however, after controlling for all other nodes in the network analysis, there were no other important edges. Relationship status indirectly influenced eHealth tools use via gender and education level. Having children was negatively linked to ePrescription use. However, parents more often used eSick leave and eReferral than childless participants.

The lack of significant relationships between residential population density and eHealth tool use indicates that barriers to access significantly diminished. The majority of participants used eHealth tools regardless of place of residence, showing that there was equal access to eHealth tool use. A fast increase in internet users, even after the pandemic (an increase of 8.5% from 2022 to 2023)^28^, might explain the widespread use of eHealth tools and reduction of barriers in rural areas.

Network analysis showed that unemployed and retired participants used public health care more, while employed participants used private health care more. Participants using public health care more often were less educated and had children, while use of paid health services was linked to higher education and being childless.

Furthermore, women were more likely to use private health care, while there was no relationship between gender and public health care use. Therefore, women were more interested in medical service use, particularly for additionally paid services. Furthermore, participants using public health services more often used ePrescription, phone consultations, and video consultations. Additionally, private health users more often used video consultations.

The strongest nodes in the network were age and employment status, along with video consultation, eSick leave, and ePrescription use. However, when analyzing expected influence, anxiety symptoms turned out to be the most influential node, along with the presence of children and eHealth tool use (mobile health app, phone consultation, IKP, EMR, eReferral, eSick leave use). It seems that the most popular eHealth tool (ePrescription) was not influential in the network. However, the presence of children was important in terms of eHealth tools use. Additionally, anxiety symptoms were the most important factor.

There are several limitations to this study. The study had a cross-sectional design, and all variables were measured at only one time point. Additionally, all measurements were based on self-assessments. Indices of physical health and economic status were not included. The UTUAT2 model was only partially referenced. The study was conducted in a specific Polish context. Future research should incorporate the full UTUAT2 model, including predictors of behavioral intentions (e.g., performance expectancy, effort expectancy), in an international longitudinal study design.

## Conclusions

When exploring eHealth, the particular eHealth tool type should be specified. Different eHealth tools are related to a variety of factors. Except for age, sociodemographic factors were not strongly related to eHealth tools use frequency. That means that social barriers to eHealth use are diminishing. The positive relationship between behavioral intentions and use behavior was confirmed for all eHealth tools, except video consultations and eSick leave. Although mental health indices were not strongly influential in the eHealth tool use network, attention should be given to anxiety levels as the factor with the highest expected influence.

## Methods

### Study design

This online cross-sectional study was conducted on 13–14 March 2023 among a nationally representative sample of 1000 Polish citizens over 18 years old. The data were collected by the Clinical Research Organization BioStat. The mean time to complete the survey was 9 min and 33 s. The participants were enrolled in a reward system (prizes). Each participant was given 40 points. There was an opportunity to exchange points for money after collecting 1000 points. The invitation to the study was sent to 130,239 users, and the first 1000 participants who met the representativity requirements (gender, place of residence, employment status) and inclusion criteria (over 18 years of age) were enrolled.

### Ethics statement

The study protocol was approved by the University Research Committee at the Lublin University of Technology, Poland, Decision No. 1/2023. The study followed the ethical requirements of anonymity and voluntariness of participation. Each person provided written informed consent. Following the Declaration of Helsinki, written informed consent was obtained from each participant before inclusion. All methods were carried out in accordance with relevant guidelines and regulations.

### Statistical analysis

The statistical analyses were performed using JASP 0.17.2.1^49^ (common method bias, correlation heatmap, network analysis), IBM SPSS 28^50^ (chi-square test, normality tests), and jamovi 2.2^51^ (descriptive statistics). A chi-square (χ^2^) test was performed for frequencies in all variables. The graphical inspection of descriptive statistics and tests of normality (Kolmogorov‒Smirnov and Shapiro‒Wilk) showed that no continuous variables met the requirements for normality of distribution. All eHealth tools use frequency indices were coded as categorical variables for the χ^2^ test. The original scale ranged from 0 = *never used* to 5 = *very often used*. The coded scale was 0 = *never used* and 1 = *used* (originally values from 1 to 5). Health care use frequency (private or public) was originally scored from 1 = *never used* to 5 = *very often used* was recoded as 0 = *never used* and 1 = *used* (originally 2–5). Behavioral intention to use eHealth tools was recoded based on the mean (11), denoting results under the mean as 0 = *low* and those over the mean as 1 = *high*. The GAD-2 and PHQ-2 scores were recoded with cutoff scores equal to or above 10^52,53^, where 0 = *no* and 1 = *yes* in terms of the presence of anxiety and depression symptoms, respectively. There is no cutoff score for the PSS-4^54^, but the recommended level for high perceived stress is 6 to 16^55^. The PSS-4 scores were recoded as 0 = *low* and 1 = *high* based on recommended values.

The next step was performing the network analysis. The Spearman’s *rho* heatmap was generated to show correlations between continuous variables. The Mann‒Whitney U test was performed to assess differences in eHealth tool use, health care use, and BI. The effect size* r* was calculated with the formula: z/√(N). According to Cohen^56^, *r* values above 0.1 can be described as small, values above 0.3 can be described as moderate, and values above 0.5 can be described as large.

Next, network analysis was performed. Network analysis is a unique method used to explore complex systems combining environmental, behavioral, and psychological data. We assumed a mutualistic model. A network is a system that consists of nodes (visually represented by circles) connected by edges (lines) that reflect the strength of the relationship between nodes, typically after controlling for all variables in the network^57^. Blue edges represent positive associations, while red edges represent negative associations. The thickness and saturation of the edges represent the strength of the relations between the nodes.

Network analysis can indicate centrality measures (betweenness, closeness, and strength). The indices are shown as standardized z scores. However, the centrality measurement is debated and faces strong criticism due to conceptual ambiguity and its adjustment in psychological networks^58,59^. In particular, betweenness and closeness centrality are incompatible as measures of node importance^59^. These two centrality measures are based on (absolute) conditional associations and do not represent physical distances, which violates the principle of transitivity^40^. However, even though the centrality measures indicate the importance of nodes to the estimated network, peripheral nodes may also be important in shaping system behavior^60^. Furthermore, a new centrality measurement, expected influence (EI), has been recommended as a more accurate measure, as it includes negative associations among nodes^45^. Expected influence successfully predicted how strongly changes in nodes were associated with changes in the remaining nodes, with higher values reflecting greater node centrality/influence^61^. Centrality strength is defined as the sum of the absolute value of all edges^62^. Therefore, centrality strength and expected influence were used in this network analysis. The remaining centrality measures, betweenness and closeness, are presented in the Supplementary Materials.

The estimator was EBICglasso with the extended Bayesian information criterion (EBIC) tuning parameter at the recommended value of 0.5 to compute a sparse Gaussian graphical model (GGM) with graphical lasso^63^. All 20 continuous, ordinal and binary variables were included in the model. Network analysis was weighted, signed, and estimated with (penalized) maximum likelihood estimation^64^. Centrality stability was calculated to assess the degree to which centrality estimates were subject to sampling error. The accuracy of the centrality indices was determined with 1000 bootstrap samples. The correlation stability (CS) coefficient should not be below 0.25 and preferably should be above 0.5. Edge stability was investigated using subset bootstrapping. The number of bootstraps was 1000, and bootstrap-type nonparametric data can be applied to continuous, categorial and ordinal data^65^.

### Sample size

G*Power^66^ was utilized to calculate an appropriate sample size. For the χ^2^ test of independence, the following parameters were used: two-tailed test, effect size of 1.5, alpha of 0.05, 95% power and proportion of discordant pairs of 0.30. The minimum sample size was determined to be 148. For network analysis, the larger the sample size is, the more stable and accurate the networks estimated. However, predicting network structure and edge weights is difficult, as little evidence exists for a priori power analysis guidance^67^. For structural equation modeling (SEM) analysis as used for common method bias, the adequate total sample size has to exceed 300 people^68^.

Sample size was calculated based on a 99% confidence level, standard deviation of 0.5, and confidence interval (margin of error) of 4.08 from the population of Polish citizens (38,000,000). The calculated sample size was 1000. Therefore, we decided to conduct a representative study among a population of 1000 participants in terms of age, gender, and employment status to enable generalization of results.

### Common method bias

To prevent common method bias (CMB), several procedures were implemented in terms of study design and data collect, e.g., different scales, mixed order of survey questions, and relatively short survey length (data collection time less than 10 min). Furthermore, the sociodemographic characteristics of the sample reflected Polish population diversification. However, all measurements were captured by the same response method at the same time point, which can be a source of CMB^69^. Therefore, to verify whether CMB significantly affected the study variables, we performed Harman’s single-factor test. All items (*N* = 23) were included in the exploratory factor analysis (EFA) with one fixed factor, a principal axis factoring extraction method and no rotation. The total variance explained was 23.87%, which is less than the threshold of 50%^69^. This indicates that there was no bias. However, this method is increasingly criticized^70,71^. Therefore, we evaluated common method bias using a common latent factor, which is proposed to be a more appropriate method^72^. Structural equation modeling (SEM) was performed with and without the common latent factor (CFL). The following step involved comparing standardized regression weights in those two models. The comparison did not reveal any significant differences between the model without CFL and with CFL, above 0.2. No items were affected by common method bias. Therefore, the research data were free of common method bias.

### Measurements

Regarding BI, the extended unified theory of acceptance and use of technology (UTAUT2)^32^ within the eHealth context^33^ was employed. However, instead of “mobile health”, we used “eHealth tools”. We added an introduction to ensure that the participants understood the notion of the eHealth tools: “eHealth is health care practice supported by electronic processes and communications. The e-health tools include: ePrescription, eSick leave, eReferral, electronic medical documentation (EMD), the Internet Patient Account (IKP), remote medical care and e-consultations (e.g., telephone consultations or video consultations), and mobile health applications.” The BI scale consists of three items evaluated on a 5-point Likert scale, varying from 1 = *strongly disagree* to 5 = *strongly agree*. The BI scale ranged from 3 to 15. The Cronbach’s α for this study was high (0.92).

The description of the eHealth Tools used in our research is presented below. An ePrescription is an electronic document replacing a prescription paper. An ePrescription can be redeemed based on a 4-digit code associated with the PESEL number of the patient to whom the ePrescription is issued. An eSick leave has replaced the traditional certificate issued in paper form by a doctor. As a result of introducing this functionality, the rest is automatically delivered to the employer and Social Security. An eReferral is another element of digitizing health care and digitizing documentation of the diagnostic and therapeutic process. Thanks to its introduction, the process of issuing a referral has been improved by eliminating potential errors (the system verifies the correctness of the e-referral) and enabling the patient to make an appointment independently. Electronic medical documentation (EMD) is created electronically with an appropriate electronic signature. An account is set up on an online platform that allows each patient to use specific digital services and collect certain medical data of a particular patient. The Internet Patient Account (IKP) stores and gives access to data medical information to the account holder or authorized person, allowing you to take care of some activities without leaving your home. Phone and video consultations are health services provided at a distance using electronic or communication systems. Mobile health applications include solutions for electronic appointments and checking test results, applications for operating medical devices (e.g., blood pressure monitor or insulin pump), or simply applications for monitoring a healthy lifestyle^35^.

eHealth tool use frequency was measured separately for each of eight tools: ePrescription, eSick leave, eReferral, electronic medical documentation (EMD), the Internet Patient Account (IKP), telephone consultation, video consultation, and mobile health applications, on a scale from 0 = n*ever* to 5 = v*ery often*. This selection of eHealth tools reflected the Polish context and was based on a review of the Polish health care system^35^.

Health care use frequency was assessed for public and private health care use on a scale ranging from 1 = n*ever* to 5 = *very often*. eHealth tool use and private/public health care use frequency were denoted as use behavior in the UTAUT2 model^32^.

The 4-item Perceived Stress Scale (PSS-4)^54,73^ was utilized to evaluate perceived stress during the last month. The items are evaluated on a scale from 0 = *never* to 4 = *very often*. PSS-4 scores range from 0 to 16. The scale’s reliability in this study was very good, with Cronbach’s α = 0.73.

The 2-item Generalized Anxiety Disorder scale (GAD-2)^52^ was utilized for anxiety assessment, while the 2-Item Patient Health Questionnaire (PHQ2)^53^ was utilized for depression assessment. The items are evaluated on scales from 0 = *not at all* to 3 = *nearly every day*. Each scale ranges from 0 to 6. Although the measurements are used worldwide and have been validated^52,53^, the internal consistency was low for anxiety and depression symptoms, and Cronbach’s α was 0.56 and 0.63 in this study, respectively, probably due to the shortening of the scales.

The sociodemographic survey included gender, age, education level, place of residence, relationship status (in a relationship vs. single), presence of children, living arrangements (living with others vs. living alone), and employment status.

### Supplementary Information

Supplementary Information.

## Data Availability

The measurements and the database are accessible at the Centre for Open Science OSF^74^.

## Abbreviations

COVID-19: The coronavirus disease 2019
UTUAT: The unified theory of acceptance and use of technology
UTUAT2: The extended theory of acceptance and use of technology
EMD: Electronic medical documentation
IKP: Internet patient account
PSS-4: The 4-item perceived stress scale
GAD-2: The 2-item generalized anxiety disorder scale
PHQ-2: The 2-item patient health questionnaire
WHO: World Health Organization
BI: Behavioral intention to use eHealth tools
