# Supplementary material for: eHealth tools use and mental health: a cross-sectional network analysis in a representative sample
Source: Sci Rep. 2024 Mar 2;14:5173. doi: 10.1038/s41598-024-55910-z (PMC10908800; doi:10.1038/s41598-024-55910-z)
Supplement: Supplementary file 1 — Supplementary Information. [file 41598_2024_55910_MOESM1_ESM.pdf]

## Supplementary Materials

### S1. Correlations

Age was significantly negatively correlated with eSick leave, video consultation and mobile health application use frequency and positively correlated with ePrescription and IKP use frequency. Age was also inversely related to private health care use, stress and depression symptoms. However, the older the participants were, the greater the behavioral intention to use eHealth tools and the more frequent the public health care use. All correlations were weak except for a very weak correlation between IKP use and public health care use.

Significant but weak positive correlations were found between place of residence and eReferral, mobile health apps, and behavioral intentions to use eHealth tools. However, there was a weak positive relationship between place of residence and private health care use. The higher the population density of the place of residence was, the more frequent the use of private health care. Due to a very weak or trivial relationship between place of residence and eHealth tool use, this variable was excluded from further statistical analysis.

Education level was significantly related to use of all eHealth tools. All relationships were weak except for a very weak relationship with video consultation use. Education level was not significantly correlated with public health care use, but there was a significant weak positive relationship with private health care use and behavioral intention to use eHealth tools.

There were significant positive moderate correlations of public health care use with use of eHealth tools (specifically, ePrescription, eReferral, IKP, and phone consultation use) and the behavioral intention to use eHealth tools. Public health care use frequency was also significantly and positively correlated with eSick leave, EMR, and video consultation use as well as perceived stress and depression symptoms, and there was a very weak correlation between public health care use frequency and anxiety symptoms. Private health care use was also significantly positively correlated with the use of all eHealth tools. The correlations of private health care use with ePrescription, eReferral, and IKP use were weak, while those with eSick leave, EMR, phone and video consultation, and mobile health app use were moderate. Private health care use was also significantly correlated with behavioral intentions to use eHealth tools and mental health indices. Those relationships were positive but weak.

The frequencies of use of all eHealth tools were significantly correlated. The highest correlations were of ePrescription use with eReferral and IKP use; eReferral use with EMR, IKP, and phone consultation use; EMR use with IKP and mobile health app use; and video consultation use with mobile health app use.

The behavioral intention to use eHealth tools (BI) was significantly positively related to not only all eHealth tools but also private and public health care use. There were high correlations of BI with ePrescription and IKP use and moderate correlations with eReferral, EMR, and phone consultation use. The remaining relationships were weak. The weakest significant correlation (< 0.1) was between BI and video consultation. BI was not significantly correlated with mental health indices.

All mental health indices were negatively linked with mental health indices, but the correlations were weak. Mental health was significantly positively related to eHealth tool use, with nonsignificant relationships of perceived stress with ePrescription and IKP use. Weak relationships were observed between perceived stress and eSick leave, phone, and video consultations use; between anxiety symptoms and eSick leave, EMR, phone and video consultations, and mobile health app use; between depression symptoms and eSick leave, eReferral, EMR, phone and video consultations, and mobile health app use.

**S2. Differences according to sociodemographic characteristics**

The results indicated that women used telephone consultations significantly more frequently ($M_{rank}$ = 521.90, $U$ = 113789.50, $p$ =.013) and video consultations less often ($M_{rank}$ = 484.52, $U$ = 116594.50, $p$ =.019) than men ($M_{rank}$ = 477.59 and $M_{rank}$ = 517.60, respectively). However, the effect sizes for telephone ($r$ = 0.08) and video consultation use ($r$ = 0.07) were very weak. Women used private health care significantly more often ($M_{rank}$ = 552.32, $U$ = 98062.50, $p$ < .001) than men, $M_{rank}$ = 445.03, with a small effect size, $r$ = 0.19. There were no other significant differences in eHealth tools and health care use or BI.

Participants in a relationship more frequently used most of the eHealth tools and public and private health care and had a higher BI than single participants. Participants in a relationship used e-Sick leave ($M_{rank}$ = 519.18, $U$ = 72119.50, $p$ < .001), eReferral ($M_{rank}$ = 515.36, $U$ = 75089.50, $p$ =.002), EMR ($M_{rank}$ = 517.11, $U$ = 73733.00, $p$ < .001), IKP ($M_{rank}$ = 515.73, $U$ = 74805.50, $p$ =.002), telephone consultation ($M_{rank}$ = 512.65, $U$ = 77196.50, $p$ =.011), and mobile health apps ($M_{rank}$ = 512.90, $U$ = 77001.00, $p$ =.007) more often than single participants, $M_{rank}$ = 435.41, $M_{rank}$ = 448.72, $M_{rank}$ = 442.64, $M_{rank}$ = 447.95, $M_{rank}$ = 453.17, $M_{rank}$ = 457.30, respectively. The effect sizes for telephone consultation ($r$ = 0.08) and mobile health app use ($r$ = 0.08) were very small, while those for eSick leave ($r$ = 0.13), eReferral ($r$ = 0.10), EMR ($r$ = 0.11), and IKP use ($r$ = 0.10) were small. There was no significant difference in video consultation use. Participants in a relationship used public ($M_{rank}$ = 512.15, $U$ = 77586.50, $p$ =.013) and private health care ($M_{rank}$ = 517.66, $U$ = 73298.50, $p$ < .001) significantly more often than single participants, $M_{rank}$ = 459.92 and $M_{rank}$ = 440.69, respectively. The effect size for public health care was very small ($r$ = 0.08), and that of private health care use was small ($r$ = 0.11). Participants in a relationship had a higher BI ($M_{rank}$ = 517.40, $U$ = 73505.00, $p$ < .001) than single participants ($M_{rank}$ = 441.62), with a small effect size, $r$ = 0.11.

Living arrangements significantly differentiated eSick leave use ($U$ = 42161.00, $p$ =.036). Participants living with others used eSick leave ($M_{rank}$ = 506.78) more frequently than those living alone ($M_{rank}$ = 448.03). Since living arrangements had a significant but very small ($r$ = 0.07) difference in only one eHealth tool, this variable was excluded from further statistical analysis.

Participants with children used eHealth tools such as eSick leave ($M_{rank}$ = 511.99, $U$ = 96748.50, $p$ =.042), eReferral ($M_{rank}$ = 523.65, $U$ = 88574.50, $p$ < .001), EMR ($M_{rank}$ = 512.28, $U$ = 96544.50, $p$ =.040), and the IKP ($M_{rank}$ = 518.28, $U$ = 92337.00, $p$ =.002) significantly more often than childless participants, $M_{rank}$ = 473.57, $M_{rank}$ = 446.24, $M_{rank}$ = 472.87, and $M_{rank}$ = 458.82, respectively. The effect sizes were very small for eSick leave ($r$ = 0.06), EMR ($r$ = 0.06), and IKP use ($r$ = 0.09) and small for eReferral use, $r$ = 0.12. Public health care was more frequently used by participants with children ($M_{rank}$ = 527.66, $U$ = 85760.50, $p$ < .001) than by those without children, $M_{rank}$ = 436.82. The effect size was small, $r$ = 0.15. BI significantly differed according to the presence of children ($U$ = 91534.50, $p$ =.001). Participants with children had a higher BI ($M_{rank}$ = 519.42) than childless participants ($M_{rank}$ = 456.13). The effect size was small, $r$ = 0.10.

Employment status significantly differentiated eHealth tools use, health care use, and BI. Employed participants used eSick leave ($M_{rank}$ = 559.06, $U$ = 51651.50, $p$ < .001), EMR ($M_{rank}$ = 512.15, $U$ = 86566.50, $p$ =.024), telephone consultation ($M_{rank}$ = 511.38, $U$ = 87137.00, $p$ =.038), video consultation ($M_{rank}$ = 519.97, $U$ = 80744.00, $p$ < .001), and mobile health apps ($M_{rank}$ = 522.10, $U$ = 79163.50, $p$ < .001) more often than unemployed participants, $M_{rank}$ = 330.26, $M_{rank}$ = 466.65, $M_{rank}$ = 468.88, $M_{rank}$ = 443.91, and $M_{rank}$ = 437.73, respectively. The strongest effect size (moderate) was for eSick leave ($r$ = 0.36). Small effect sizes were found for video consultation ($r$ = 0.15) and mobile health app use ($r$ = 0.14). The remaining effect sizes were very small. Employment status did not differentiate ePrescription, eReferral, or IKP use. Employed participants used public ($M_{rank}$ = 533.78, $U$ = 86713.00, $p$ =.025) and private health care ($M_{rank}$ = 545.22, $U$ = 61964.00, $p$ < .001) more than unemployed

participants, $M_{rank}$ = 489.05, and $M_{rank}$ = 370.55, respectively. The effect size for public health care use was very small ($r$ = 0.07), and for private health care use, the effect size was small, $r$ = 0.27. Additionally, BI was higher in employed participants ($M_{rank}$ = 512.94, $U$ = 85974.00, $p$ =.019) than in unemployed participants ($M_{rank}$ = 464.34). Nevertheless, the effect size was very small, $r$ = 0.07.

Table S1. Weights matrix for estimated network of eHealth tools use

| | Network | | | | | | | | | | | | | | | | | | | |
|---|---|---|---|---|---|---|---|---|---|---|---|---|---|---|---|---|---|---|---|---|
| **Variable** | **Gender** | **Age** | **Edu** | **RS** | **Children** | **Employment** | **BI** | **ePrescr** | **eSick** | **eReferr** | **EMR** | **IKP** | **Phone** | **Video** | **App** | **Stress** | **Anxiety** | **Depression** | **Public** | **Private** |
| Gender | 0.000 | -0.404 | 0.034 | 0.064 | 0.152 | -0.049 | -0.053 | 0.000 | -0.069 | 0.000 | 0.000 | 0.000 | 0.135 | -0.240 | 0.014 | 0.000 | 0.086 | 0.053 | 0.000 | 0.206 |
| Age | -0.404 | 0.000 | 0.000 | -0.136 | 0.423 | -0.093 | 0.031 | **0.123** | **-0.234** | 0.015 | 0.039 | 0.037 | 0.031 | -0.096 | -0.023 | -0.054 | -0.003 | 0.028 | 0.009 | 0.000 |
| Edu | 0.034 | 0.000 | 0.000 | 0.075 | 0.019 | 0.223 | 0.011 | 0.072 | -0.076 | 0.074 | 0.000 | 0.098 | 0.000 | -0.016 | 0.000 | 0.000 | 0.024 | -0.012 | **-0.135** | 0.098 |
| RS | 0.064 | -0.136 | 0.075 | 0.000 | 0.566 | 0.130 | 0.019 | 0.000 | -0.014 | 0.000 | 0.040 | 0.003 | 0.000 | -0.010 | 0.000 | -0.022 | 0.000 | -0.041 | 0.011 | 0.071 |
| Children | 0.152 | 0.423 | 0.019 | 0.566 | 0.000 | 0.047 | 0.000 | -0.085 | 0.058 | 0.058 | 0.000 | 0.000 | 0.000 | 0.002 | 0.000 | 0.000 | 0.000 | -0.044 | **0.130** | **-0.118** |
| Employment | -0.049 | -0.093 | 0.223 | 0.130 | 0.047 | 0.000 | 0.077 | **-0.143** | **0.532** | **-0.120** | -0.091 | -0.062 | -0.029 | 0.000 | 0.000 | -0.034 | -0.013 | 0.000 | **-0.125** | **0.195** |
| BI | -0.053 | 0.031 | 0.011 | 0.019 | 0.000 | 0.077 | 0.000 | **0.203** | 0.000 | 0.041 | 0.008 | **0.232** | **0.110** | **-0.177** | **0.147** | -0.018 | 0.000 | 0.000 | 0.058 | 0.048 |
| ePrescr | 0.000 | **0.123** | 0.072 | 0.000 | -0.085 | -0.143 | 0.203 | 0.000 | 0.136 | 0.314 | 0.013 | 0.169 | 0.141 | -0.191 | 0.029 | **-0.013** | 0.000 | 0.009 | **0.171** | 0.036 |
| eSick | -0.069 | **-0.234** | -0.076 | -0.014 | 0.058 | 0.532 | 0.000 | 0.136 | 0.000 | 0.252 | 0.114 | 0.037 | 0.061 | 0.134 | 0.035 | **0.026** | 0.003 | 0.000 | 0.073 | 0.000 |
| eReferr | 0.000 | 0.015 | 0.074 | 0.000 | 0.058 | -0.120 | 0.041 | 0.314 | 0.252 | 0.000 | 0.177 | 0.103 | 0.118 | 0.000 | 0.000 | 0.000 | 0.000 | 0.000 | 0.031 | 0.000 |
| EMR | 0.000 | 0.039 | 0.000 | 0.040 | 0.000 | -0.091 | 0.008 | 0.013 | 0.114 | 0.177 | 0.000 | 0.278 | 0.029 | 0.182 | 0.239 | -0.009 | 0.000 | 0.000 | -0.019 | 0.042 |
| IKP | 0.000 | 0.037 | 0.098 | 0.003 | 0.000 | -0.062 | 0.232 | 0.169 | 0.037 | 0.103 | 0.278 | 0.000 | 0.056 | 0.000 | 0.049 | 0.000 | 0.000 | -0.003 | 0.075 | 0.000 |
| Phone | **0.135** | 0.031 | 0.000 | 0.000 | 0.000 | -0.029 | 0.110 | **0.141** | 0.061 | 0.118 | 0.029 | 0.056 | 0.000 | 0.215 | 0.068 | **0.012** | **0.026** | 0.000 | 0.112 | 0.041 |
| Video | **-0.240** | -0.096 | -0.016 | -0.010 | 0.002 | 0.000 | -0.177 | **-0.191** | 0.134 | 0.000 | 0.182 | 0.000 | 0.215 | 0.000 | 0.425 | **0.027** | 0.000 | **0.036** | 0.121 | 0.207 |
| App | 0.014 | -0.023 | 0.000 | 0.000 | 0.000 | 0.000 | 0.147 | 0.029 | 0.035 | 0.000 | 0.239 | 0.049 | 0.068 | 0.425 | 0.000 | **-0.025** | 0.006 | **0.028** | -0.050 | 0.016 |
| Stress | 0.000 | -0.054 | 0.000 | -0.022 | 0.000 | -0.034 | -0.018 | -0.013 | 0.026 | 0.000 | -0.009 | 0.000 | 0.012 | 0.027 | -0.025 | 0.000 | 0.427 | 0.157 | 0.057 | 0.000 |
| Anxiety | 0.086 | -0.003 | 0.024 | 0.000 | 0.000 | -0.013 | 0.000 | 0.000 | 0.003 | 0.000 | 0.000 | 0.000 | 0.026 | 0.000 | 0.006 | 0.427 | 0.000 | 0.560 | 0.000 | 0.053 |
| Depression | 0.053 | 0.028 | -0.012 | -0.041 | -0.044 | 0.000 | 0.000 | 0.009 | 0.000 | 0.000 | 0.000 | -0.003 | 0.000 | 0.036 | 0.028 | 0.157 | 0.560 | 0.000 | 0.000 | 0.000 |
| Public | 0.000 | 0.009 | -0.135 | 0.011 | 0.130 | -0.125 | 0.058 | **0.171** | 0.073 | 0.031 | -0.019 | 0.075 | **0.112** | **0.121** | -0.050 | **0.057** | 0.000 | 0.000 | 0.000 | 0.000 |
| Private | **0.206** | 0.000 | 0.098 | 0.071 | -0.118 | 0.195 | 0.048 | 0.036 | 0.000 | 0.000 | 0.042 | 0.000 | 0.041 | 0.207 | 0.016 | 0.000 | **0.053** | 0.000 | 0.000 | 0.000 |

*Note*. ePresr = ePrescription; eSick = eSick leave; eReferr = eReferral; EMR = electronic medical records; IKP = Internet Patient Account; Phone = telephone consultation; Video = video consultation; App = mobile health application. Health care use: Public = public health care use; Private = private health care use frequency; BI = behavioral intention to use eHealth tools. The dichotomized sociodemographic nodes are as follows: Gender (women = 1), Children (presence of children, yes = 1), RS = relationship status (in a relationship= 1), Education (tertiary education and higher= 1), Employment (employed =1).

Table S2. Centrality measures per variable

| | Network | | | |
|---|---|---|---|---|
| **Variable** | **Betweenness** | **Closeness** | **Strenght** | **Expected influence** |
| Gender | 2.494 | 1.380 | 0.481 | -2.040 |
| Age | 1.615 | 1.215 | 1.088 | -2.625 |
| Edu | -0.747 | -0.770 | -1.154 | -0.655 |
| RS | -0.747 | -0.304 | -0.505 | 0.004 |
| Children | 0.187 | 0.280 | 0.881 | 1.120 |
| Employment | 0.077 | 0.472 | 1.609 | -0.762 |
| BI | -0.747 | -0.212 | -0.414 | -0.039 |
| ePrescr | -0.033 | 0.670 | 1.292 | 0.572 |
| eSick | 0.736 | 0.890 | 1.300 | 0.778 |
| eReferr | -0.582 | 0.284 | -0.224 | 0.764 |
| EMR | -0.253 | -0.121 | -0.284 | 0.715 |
| IKP | -0.582 | -0.693 | -0.502 | 0.791 |
| Phone | -0.747 | -0.152 | -0.554 | 0.919 |
| Video | 1.780 | 1.489 | 1.925 | -0.335 |
| App | -0.747 | 0.449 | -0.633 | 0.505 |
| Stress | -0.637 | -1.935 | -1.392 | -0.553 |
| Anxiety | 1.011 | -1.626 | -0.504 | 1.024 |
| Depression | -0.747 | -1.912 | -1.141 | 0.046 |
| Public | -0.582 | 0.063 | -0.569 | -0.582 |
| Private | -0.747 | 0.531 | -0.699 | 0.349 |

*Note*. ePresr = ePrescription; eSick = eSick leave; eReferr = eReferral; EMR = electronic medical records; IKP = Internet Patient Account; Phone = telephone consultation; Video = video consultation; App = mobile health application. Health care use: Public = public health care use; Private = private health care use frequency; BI = behavioral intention to use eHealth tools. The dichotomized sociodemographic nodes are as follows: Gender (women = 1), Children (presence of children, yes = 1), RS = relationship status (in a relationship= 1), Education (tertiary education and higher= 1), Employment (employed =1).

Table S3. Clustering measures per variable

| Variable | Network | | | |
|---|---|---|---|---|
| | **Barrat** | **Onnela** | **WS** | **Zhang** |
| Gender | 0.231 | 0.798 | -0.381 | -0.210 |
| Age | -0.673 | -0.287 | -0.655 | 0.013 |
| Children | 0.421 | 0.996 | 0.176 | -0.392 |
| Edu | -0.402 | -0.757 | -1.464 | 0.067 |
| ePrescr | 0.276 | 0.927 | -0.220 | -0.670 |
| eSick | 0.504 | 0.609 | -0.600 | -0.346 |
| eReferr | 2.059 | 2.233 | 2.727 | 0.519 |
| EMR | 0.341 | -0.159 | 1.536 | -0.617 |
| IKP | 1.144 | 0.297 | 0.786 | -0.741 |
| Phone | -0.043 | 0.029 | 0.186 | 0.230 |
| Video | 0.206 | 0.865 | -0.031 | -1.393 |
| App | 0.716 | -0.597 | -0.214 | 0.678 |
| Public | 0.528 | 0.007 | 1.053 | -0.602 |
| Private | -0.014 | 0.141 | -0.858 | -0.737 |
| RS | -0.276 | -0.945 | -0.464 | 1.024 |
| Stress | -1.770 | -1.656 | 0.786 | 2.377 |
| Anxiety | -1.556 | -1.576 | -0.537 | -0.350 |
| Depression | -2.340 | -1.546 | -1.410 | 2.367 |
| BI | 0.376 | -0.217 | 0.186 | -0.108 |
| Employment | 0.275 | 0.840 | -0.600 | -1.111 |

*Note*. ePresr = ePrescription; eSick = eSick leave; eReferr = eReferral; EMR = electronic medical records; IKP = Internet Patient Account; Phone = telephone consultation; Video = video consultation; App = mobile health application. Health care use: Public = public health care use; Private = private health care use frequency; BI = behavioral intention to use eHealth tools. The dichotomized sociodemographic nodes are as follows: Gender (women = 1), Children (presence of children, yes = 1), RS = relationship status (in a relationship= 1), Education (tertiary education and higher= 1), Employment (employed =1).
